# Toxicokinetic/Toxicodynamic Interaction Studies in Rats between the Drugs of Abuse γ-Hydroxybutyric Acid and Ketamine and Treatment Strategies for Overdose

**DOI:** 10.3390/pharmaceutics13050741

**Published:** 2021-05-18

**Authors:** Nisha V. Kwatra, Marilyn E. Morris

**Affiliations:** 1 Department of Pharmaceutical Sciences, School of Pharmacy and Pharmaceutical Sciences, State University of New York, Buffalo, NY 14214, USA; Nisha.Kwatra@fda.hhs.gov; 2 Division of Inflammation and Immune Pharmacology, Office of Clinical Pharmacology, Office of Translational Sciences, Center for Drug Evaluation and Research, Food and Drug Administration, Silver Spring, MD 20993, USA

**Keywords:** GHB, monocarboxylate transporter, ketamine, toxicity, respiratory depression, sedation, lethality, drug-drug interaction

## Abstract

γ-hydroxybutyric acid (GHB) is widely abused alone and in combination with other club drugs such as ketamine. GHB exhibits nonlinear toxicokinetics, characterized by saturable metabolism, saturable absorption and saturable renal reabsorption mediated by monocarboxylate transporters (MCTs). In this research, we characterized the effects of ketamine on GHB toxicokinetics/toxicodynamics (TK/TD) and evaluated the use of MCT inhibition and specific receptor antagonism as potential treatment strategies for GHB overdose in the presence of ketamine. Adult male Sprague-Dawley rats were administered GHB 600 mg/kg i.v. alone or with ketamine (6 mg/kg i.v. bolus plus 1 mg/kg/min i.v. infusion). Plasma and urine samples were collected and respiratory parameters (breathing frequency, tidal and minute volume) continuously monitored using whole-body plethysmography. Ketamine co-administration resulted in a significant decrease in GHB total and metabolic clearance, with renal clearance remaining unchanged. Ketamine prevented the compensatory increase in tidal volume produced by GHB, and this resulted in a significant decline in minute volume when compared to GHB alone. Sleep time and lethality were also increased after ketamine co-administration when compared to GHB. L-lactate and AR-C155858 (potent MCT inhibitor) treatment resulted in an increase in GHB renal and total clearance and improvement in respiratory depression. AR-C155858 administration also resulted in a significant decrease in GHB brain/plasma ratio. SCH50911 (GABA_B_ receptor antagonist), but not naloxone, improved GHB-induced respiratory depression in the presence of ketamine. In conclusion, ketamine ingestion with GHB can result in significant TK/TD interactions. MCT inhibition and GABA_B_ receptor antagonism can serve as potential treatment strategies for GHB overdose when it is co-ingested with ketamine.

## 1. Introduction

γ-hydroxybutyric acid (GHB, with the street name of Liquid Ecstasy) is a recreational drug that is widely abused for its euphotic effects at nightclubs and raves. It was reported in 2011 by Substance Abuse and Mental Health Services Administration that emergency department visits resulting from GHB overdose in the United States range between 1000–2000 annually. Adverse effects resulting from GHB overdose include hypothermia, respiratory depression, coma and death [1,2,3]. In typical cases of overdose, GHB is usually not ingested alone. In majority of cases, GHB is found to be co-ingestion either with ethanol and/or other club drugs [3,4,5]. Significant toxicodynamic interactions using multiple toxicodynamic endpoints (such as sedation, respiratory depression and lethality) have been reported between GHB and ethanol [6,7]. However, our knowledge concerning the interactions between GHB and other commonly co-ingested club drugs such as ketamine, 3, 4-methylenedioxymethamphetamine (MDMA), and rohypnol is limited. Ketamine was reported to be second most abused club drug besides GHB [8] and between 2006 and 2010 the number of persons reporting the consumption of GHB and ketamine has increased significantly [9]. Ketamine is a dissociative anesthetic with a high abuse liability and can also lead to respiratory depression at high doses, as seen in overdose situations. A recent survey of 131 GHB users showed that ketamine was co-ingested by 30% of the individuals and the risk of hospital treatment increased among GHB users following ketamine co-ingestion [4].

Although abuse of GHB, alone and with other club drugs, has been recognized as a significant issue in public health, there is currently no approved antidote for GHB overdose and treatment is limited to supportive care. GHB exhibits nonlinear toxicokinetics, characterized by saturable metabolism, saturable oral absorption and saturable renal reabsorption [10,11,12]. GHB has been reported to be a substrate for monocarboxylate transporters (MCTs) in organs such as the liver, kidney, intestine, and brain [13,14,15,16,17]. MCT inhibition has been evaluated in our laboratory as a potential treatment strategy for GHB overdose through the inhibition of active renal reabsorption of GHB mediated by MCTs [11,18,19]. This strategy is useful because renal clearance represents a major pathway of GHB elimination at higher concentrations, as found in overdose situations [11]. We have demonstrated that renal and total clearance of GHB can be increased by co-administration of MCT inhibitors with GHB in rats [18]. MCT inhibition also results in both a decrease in sedative/hypnotic effects of GHB as well as improves respiratory depression [18,19]. Recently, we have also shown that higher doses of the MCT inhibitor, L-lactate can decrease GHB concentrations in brain extracellular fluid of rats demonstrating that MCT inhibition can block the uptake of GHB into the brain which is its site of action [20]. However, the potential efficacy of MCT inhibition as a strategy for the treatment of GHB overdose in the presence of ketamine needs to be evaluated.

GHB is known to have binding affinity towards both GHB and GABA_B_ receptors. The pharmacological effects of GHB such as sedation, hypothermia and respiratory depression are known to be mediated by its binding to GABA_B_ receptors in the brain [19,21,22]. An improvement in these toxicodynamic end points has been demonstrated following treatment with GABA_B_ receptor antagonists [19,21]. Ketamine (a non-competitive *N*-methyl-d-aspartate receptor (NMDA) receptor antagonist) which accounts for most of its anesthetic effects. Ketamine produces dose-dependent sedation after intravenous as well as oral administration in rats, with mechanisms different than that of GHB [23,24]. Intraperitoneal administration of ketamine has been shown to cause significant respiratory depression in mice which was completely abolished in μ-opioid receptor knockout mice [25]. Measurement of respiration in human volunteers after intravenous ketamine administration also showed a log-linear dose related depression [26]. This suggests that ketamine produces respiratory depression through mechanisms different than that of GHB. Recent studies have shown that NMDA receptor antagonists such as ketamine and phencyclidine can enhance GHB-mediated cataleptic effects in mice [27]. Ketamine has also shown to potentiate the respiratory depression induced by opioids when administered at subanesthetic doses in rats [28].

**Figure 1:**
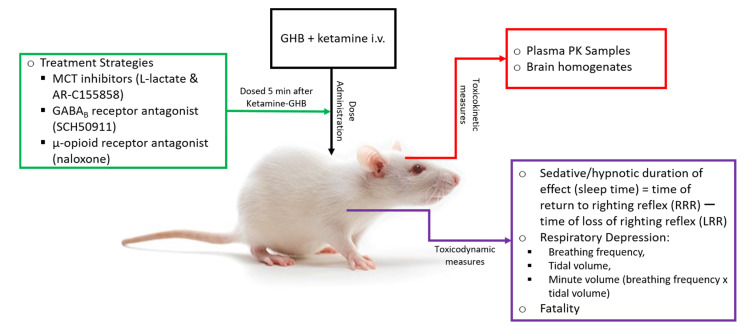
Schematic of Study Design.

Although it is known that GHB is commonly co-ingested with ketamine in a recreational setting, the toxicokinetic/toxicodynamic (TK/TD) interactions between these club drugs using clinically relevant end points currently remain unknown. Therefore, the first objective the study was to characterize the effects of ketamine on TK/TD of GHB by using the end points of sedation, respiratory depression, and fatality. The second objective was to evaluate the use of potential treatment strategies including MCT inhibition, GABA_B_ receptor and μ-opioid receptor antagonism, as potential treatment strategies for GHB overdoses in the presence of ketamine. The summary of the experimental strategy is presented in Figure 1.

## 2. Materials and Methods

### 2.1. Chemicals and Reagents

Sodium GHB used in these studies was provided by the National Institute on Drug Abuse. (±) Ketamine Hydrochloride, sodium L-lactate, naloxone hydrochloride dihydrate, staurosporine, and phorbol 12-myristate 13-acetate were purchased from Sigma-Aldrich (St. Louis, MO, USA). Deuterated GHB (GHB-d_6_) was purchased from Cerilliant Corporation (Round Rock, TX, USA). (2S)-(+)-5,5-Dimethyl-2-morpholineacetic acid (SCH50911) was purchased from R&D Systems (Minneapolis, MN, USA). High-performance liquid chromatography grade acetonitrile and acetic acid were purchased from Honeywell Burdick & Jackson (Muskegon, MI, USA). 6-[(3,5-Dimethyl-1H-pyrazol-4-yl) methyl]-5-[[(4S)-4-hydroxy-2-isoxazolidinyl]carbonyl]-3-methyl-1-(2-methylpropyl)thieno [2,3-d]pyrimidine-2,4(1H,3H)-dione (AR-C155858) was purchased from Chemscene (Monmouth Junction, NJ, USA).

### 2.2. Animals and Surgery

Male Sprague-Dawley rats (Harlan, Indianapolis, IN, USA) weighing 270 to 330 g were used for all experiments. All animal procedures were approved by the University at Buffalo Institutional Animal Care and Use Committee. The study was conducted according to the guidelines of the Declaration of Helsinki, and ap-proved by the Institutional Review Board of the University at Buffalo (PROTO201800102 approved 10/14/18). Animals were housed under controlled temperature and humidity with an artificial 12-h light/dark cycle. Food was available ad libitum. Jugular and femoral vein cannulae were surgically implanted under anesthesia with ketamine/xylazine (75/10 mg/kg). Cannulae were flushed daily with 40 IU/mL heparinized saline to maintain patency. Rats were allowed at least 72 h for recovery from surgery before the first experimental day.

### 2.3. Toxicokinetic/Toxicodynamic Interaction Studies

#### 2.3.1. Effect of Ketamine on the Sedative Effects of GHB

The effect of ketamine on the sedative effects of GHB was measured using the endpoint of righting reflex, similar to our previous studies [18,29]. Rats were administered with GHB 400 mg/kg i.v. with ketamine 6 mg/kg or 20 mg/kg via the jugular vein cannula (*n* = 5 for GHB 400 mg/kg, *n* = 4 for GHB 400 mg/kg + ketamine 6 mg/kg, *n* = 4 for GHB 400 mg/kg + ketamine 20 mg/kg) in each treatment group). This experiment was performed at a similar time and in a similar manner to our previous study assessing sedative effects of GHB alone [29]; therefore, data from rats administered GHB 600 mg/kg alone data were used from the previous publication for comparison purposes. The sedative/hypnotic duration of effect (sleep time) was measured as the difference between the time of loss-of-righting reflex (LRR) and time of return-to-righting reflex (RRR). LRR and RRR are defined as the time at which the animal lost or regained the ability to right itself when placed on its back. The animals were euthanized at RRR under isoflurane anesthesia followed by collection of blood and brain samples. Brain samples were immediately frozen in liquid nitrogen and stored at −80 °C until analysis. In these studies, GHB was administered as a 200 mg/mL solution in sterile water and ketamine as a 5 mg/mL solution in normal saline.

#### 2.3.2. Effect of Ketamine on GHB Toxicokinetics, GHB-Induced Respiratory Depression, and Fatality

The effect of ketamine on GHB-induced respiratory depression was studied using whole-body plethysmography similar to our earlier studies [19]. Animals were placed in plethysmography chambers 1 h prior to drug administration for acclimatization to the chambers for 45 min before five baseline recordings were collected over 15 min. To evaluate the effect of ketamine on GHB TK and GHB-induced respiratory depression, GHB 600 mg/kg i.v. was administered via the jugular vein cannula alone (*n* = 5) or in combination with ketamine (6 mg/kg i.v. bolus 8 min before GHB administration, followed by 1 mg/kg/min i.v. infusion for 60 min) (*n* = 6). Using this dosing regimen of ketamine, steady-state concentrations of ketamine were rapidly achieved. In all the animal groups, GHB administration was considered time 0 and respiratory parameters, breathing frequency, tidal volume, and minute volume (breathing frequency x tidal volume) were recorded at 2.5, 5, 7.5, 10, 15, 20, 25, and 30 min and every 15 min thereafter until 6 h. In all groups of animals, blood and urine samples were collected for 6 h after GHB administration. GHB was administered as a 300 mg/mL solution in sterile water via the jugular vein cannula. The ketamine bolus was administered as a 5 mg/mL solution in normal saline via the jugular vein cannula and ketamine infusion as a 10 mg/mL solution in normal saline via the femoral vein cannula. To assess the effect of ketamine on GHB-associated fatality and the effects of potential treatment strategies for preventing fatality due to respiratory arrest in GHB-ketamine intoxication, GHB (400 mg/kg i.v. bolus followed by a 208 mg/kg/h iv infusion) was administered alone or in combination with ketamine (6 mg/kg i.v. bolus followed by a 1 mg/kg/min i.v. infusion) (*n* = 8 in each treatment group). Ketamine was also administered alone at a similar dose in a separate group of animals. The GHB bolus was administered as a 5 mg/mL solution in sterile water via the jugular vein cannula and GHB infusion was administered as a 16.5 mg/mL solution in sterile water via the femoral vein cannula.

#### 2.3.3. Effect of Ketamine on GHB Brain Concentrations

To assess the effect of ketamine on GHB brain concentrations, GHB (400 mg/kg i.v. bolus followed by a 208 mg/kg/h i.v. infusion) was administered alone or in combination with ketamine (6 mg/kg i.v. bolus followed by a 0.1 mg/kg/min (low dose) or 0.287 mg/kg/min (medium dose) i.v. infusion) (*n* = 7 GHB alone, *n* = 4 for GHB + low dose ketamine, *n* = 4 for GHB + medium dose ketamine). The GHB dose was selected to produce steady-state GHB plasma concentrations of 800 µg/mL, as this is similar to the high concentrations of GHB observed in rats after the 600 mg/kg GHB i.v. dose used in the TK study above. The animals were euthanized at 4 h post-GHB-ketamine administration under isoflurane anesthesia followed by collection of blood and brain samples at steady state. Brain samples were immediately frozen in liquid nitrogen and stored at −80 °C until analysis.

### 2.4. Potential Treatment Strategies for Overdose

#### 2.4.1. Effect of MCT Inhibition on the Sedative Effects of GHB

To assess MCT inhibition as a potential treatment strategy for improving sedation in GHB-ketamine overdoses, the MCT inhibitor L-lactate (66 mg/kg i.v. bolus followed by a 302.5 mg/kg/h i.v. infusion) or AR-C155858 (1 mg/kg i.v. bolus) was administered 5 min after GHB-ketamine and sleep time was measured in each group (*n* = 4 for GHB + Ketamine, *n* = 3 for GHB + Ketamine + AR-C155858, *n* = 4 for GHB + Ketamine + L-lactate). This dose of L-lactate was chosen to increase plasma L-lactate concentrations by 1–2 mM [19]. L-Lactate was administered as a 40 mg/mL solution in sterile water via the femoral vein cannula. AR-C155858 was administered as a 2.5 mg/mL solution in 10% cyclodextrin in normal saline.

#### 2.4.2. Effect of Treatment Strategies on GHB Toxicokinetics, GHB-Induced Respiratory Depression, and Fatality

The effect of potential treatment strategies on GHB-induced respiratory depression in the presence of ketamine was studied using whole-body plethysmography similar to the studies described above. The different treatments were administered intravenously 5 min after GHB-ketamine administration. Treatment strategies included MCT inhibitors, L-lactate (66 mg/kg bolus followed by 302.5 mg/kg/h infusion for 6 h) (*n* = 4) or AR-C155858 (1 mg/kg i.v. bolus) (*n* = 4), GABA_B_ receptor antagonist SCH50911 (10 mg/kg i.v. bolus) (*n* = 3), and µ-opioid receptor antagonist naloxone (2 mg/kg i.v. bolus) (*n* = 3). In an additional group of animals, the effect of the combination of SCH50911 and naloxone (*n* = 4) was also assessed. All the treatment groups were compared with the GHB plus ketamine group (*n* = 6) to determine the effects of treatment on GHB-induced respiratory depression in the presence of ketamine. In these experiments, SCH50911 was administered as a 10 mg/mL solution in saline and naloxone as a 1 mg/mL solution in saline via the jugular vein cannula. To assess the effects of potential treatment strategies on the fatality associated with the combination of GHB and ketamine, L-lactate (66 mg/kg i.v. bolus and 302.5 mg/kg/h i.v. infusion (low dose) and 605 mg/kg/h i.v. infusion (high dose)) and AR-C155858 (1 mg/kg i.v. bolus) were administered 5 min after GHB-ketamine administration. The doses of L-lactate were chosen to increase plasma lactate concentrations by 1–2 mM (low dose) and above 4 mM (high dose), respectively (*n* = 8 in each treatment group). The number of animals that survived in each treatment group was observed. Animals were pronounced dead when respiration ceased for several minutes. To assess the effects of MCT inhibition on GHB brain/plasma partitioning in the presence of ketamine, L-lactate (66 mg/kg bolus and 302.5 mg/kg/h infusion) (*n* = 4) or AR-C155858 (1 mg/kg bolus) (*n* = 3) were administered 5 min after GHB-ketamine administration. The animals were euthanized at 4 h and brain and plasma samples obtained as described above.

### 2.5. Sample Analysis

GHB plasma concentrations were measured using a modification of the previously published LC/MS/MS assay [19,29]. For the samples containing both GHB and ketamine, this method was modified and validated for the measurement of plasma ketamine concentrations. Plasma samples were prepared by adding 5 µL of internal standard solution containing GHB-d_6_ (125 µg/mL) and ketamine-d_4_ (500 ng/mL) to 50 µL of sample. Plasma standards and quality controls were prepared by adding 5 µL of internal standard solution containing GHB-d_6_ (125 µg/mL) and ketamine-d_4_ (500 ng/mL) and 5 µL of stock solution containing both GHB and ketamine to 45 µL of blank plasma. To precipitate the plasma proteins, 800 µL of 0.1% formic acid in acetonitrile was added. The samples were vortexed and then centrifuged at 10,000× *g* for 20 min at 4 °C. An aliquot (750 µL) of the supernatant was withdrawn and evaporated under a stream of nitrogen gas. Samples were reconstituted in 250 µL of aqueous mobile phase.

All LC/MS/MS analyses were performed on an Agilent 1100 series HPLC with an online degasser, binary pump and autosampler (Agilent Technologies, Palo Alto, CA, USA) linked to a PE Sciex API triple-quadrupole tandem mass spectrometer with a turbo ion spray (Applied Biosystems, Foster City, MA, USA) was used. HPLC conditions and mass spectrometer parameters are detailed in [19]. Regression analysis of peak area ratios of GHB/GHB-d_6_ and ketamine/ketamine-d_4_ was used to assess linearity of the curve. The intra-day and inter-day precision and accuracy were determined using quality control (QC) samples at 10 µg/mL (low QC), 125 µg/mL (medium QC), and 400 µg/mL (high QC) for GHB and at 20 ng/mL (low QC), 500 ng/mL (medium QC), and 1500 ng/mL (high QC) for ketamine. For determination of the intra-day precision and accuracy, quality control samples were analyzed in triplicate on each day, whereas for the inter-day precision and accuracy, quality control samples were analyzed on three different days. The precision was determined by the coefficient of variation, and accuracy was measured by comparing the calculated concentration with the known concentration. GHB concentrations in urine were measured using a previously described LC-MS/MS method [29].

### 2.6. Data/Statistical Analysis

GHB TK parameters were determined by noncompartmental analysis (WinNonlin 5.2 software, Pharsight, Palo Alto, CA, USA). Area under the plasma concentration-time curve (AUC) was determined using the trapezoidal method. Total clearance (CL) was determined as dose/AUC. Renal clearance (CL_R_) was determined as A_e_/AUC, where Ae is the total amount of GHB excreted in the urine. Metabolic or nonrenal clearance (CL_m_) was determined as CL–CL_R_. TD descriptors for respiratory depression were determined: area under the effect curve (AUEC), maximum effect (E_max_) and duration of effect (T_d_). AUEC was determined using WinNonlin software. For the determination of statistically significant differences in TK/TD parameters, mean values were compared using a Student *t*-test or one-way analysis of variance followed by a Tukey’s post-hoc test. A *p*-value < 0.05 was considered statistically significant.

### 2.7. GHB Cell Uptake Studies

The immortalized rat brain capillary endothelial cell line (RBE4) was kindly provided by Professor P. Couraud (Institut Cochin, Paris, France). RBE4 cells (passages 39–44) were used in all studies, as described previously [20]. Briefly, cells were grown at 37 °C with 5% CO_2_ on Type-I collagen-coated flasks, and media was changed every 2–3 days. The culture medium was 1:1 α-minimum essentials medium/Hams F10 nutrient mixture supplemented with L-glutamine (2.0 mM), geneticin (300 μg/mL), human recombinant fibroblast growth factor (1 ng/mL), gentamicin (50 μg/mL), and 10% *v*/*v* fetal bovine serum (FBS). Cells were passaged using 0.25% trypsin/EDTA and seeded on individual 35 × 10 mm collagen-coated wells for uptake studies which were performed 7 days post-confluence (the time needed for formation of tight junctions).

To study the effect of ketamine on GHB uptake, cells were washed and equilibrated for 20 min at 37 °C with uptake buffer containing 138 mM NaCl, 1.8 mM CaCl_2_, 5.4 mM KCl, 0.8 mM MgSO_4_, 1.0 mM Na_2_HPO_4_, 5.5 mM d-glucose, and 20 mM HEPES (pH 7.4). Cells were then equilibrated to room temperature for 5 min followed by incubation with 10 mM [^3^H]-GHB alone or with 5, 15, or 40 µM ketamine for 15 s. Previous work indicated that 15 s is within the time of linear uptake of GHB in RBE4 cells [15]. In another set of experiments, cells were pre-incubated with ketamine (5, 15, or 40 µM) at 37 °C for 2 h before incubation with 10 mM [^3^H]-GHB for 15 s to assess the effect of ketamine pre-incubation on GHB uptake. In addition, the effect of various pre-incubation times with ketamine (0.5, 2, 4, 8 and 24 h) was also studied in these cells. A concentration of 10 mM GHB was used in these studies since it is similar to the targeted steady-state concentrations in rats in the in vivo studies. Ketamine concentrations were also selected to reflect the steady-state concentrations of ketamine as observed in vivo in the above studies. The effect of a protein kinase C (PKC) activator, phorbol 12-myristate 13-acetate (PMA) and the effect of PKC inhibitor on ketamine-induced changes in GHB uptake were also studied. Cells were pre-incubated with PMA (100 nM) or ketamine (40 µM) for 2 h in the presence and absence of a PKC inhibitor, staurosporine, before incubation with 10 mM [^3^H]-GHB for 15 s. After incubation with [^3^H]-GHB, cells were washed 3 times with ice-cold uptake buffer and lysed with 0.5 mL 1.0 N NaOH for 60 min at room temperature. After cell lysis, NaOH was neutralized with 0.5 mL 1.0 N HCl. [^3^H]-GHB uptake into the cells was determined by liquid scintillation counting and normalized by protein amounts (mg) measured using bicinchoninic acid assay (Pierce, Rockford, IL, USA) with bovine serum albumin as a standard.

## 3. Results

### 3.1. Effect of Ketamine on GHB Toxicokinetics/Toxicodynamics

#### 3.1.1. Effect of Ketamine on GHB Toxicokinetics

**Figure 2:**
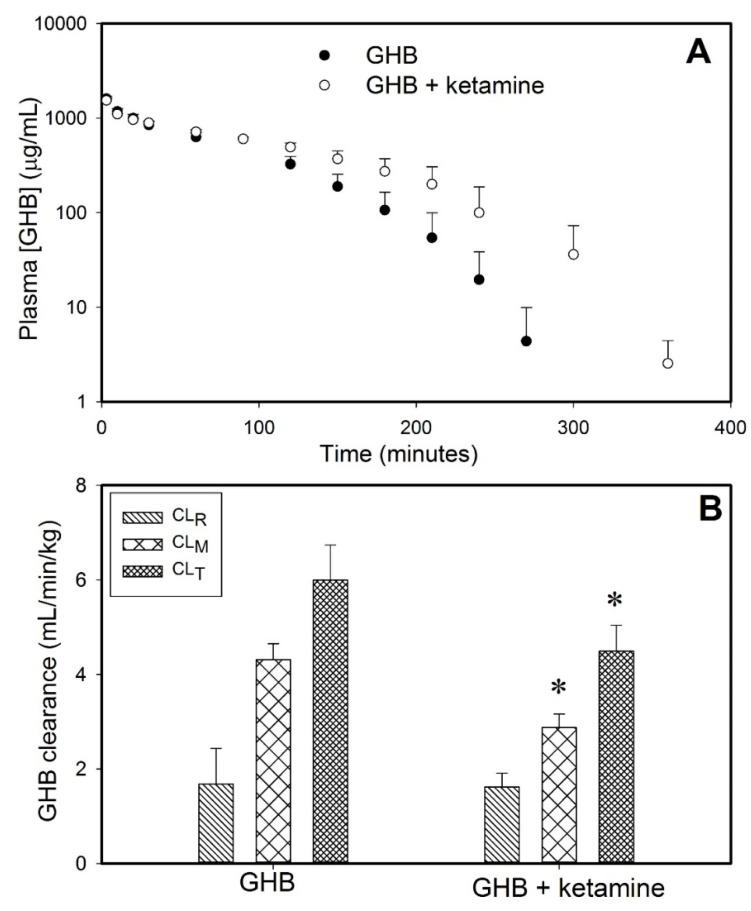

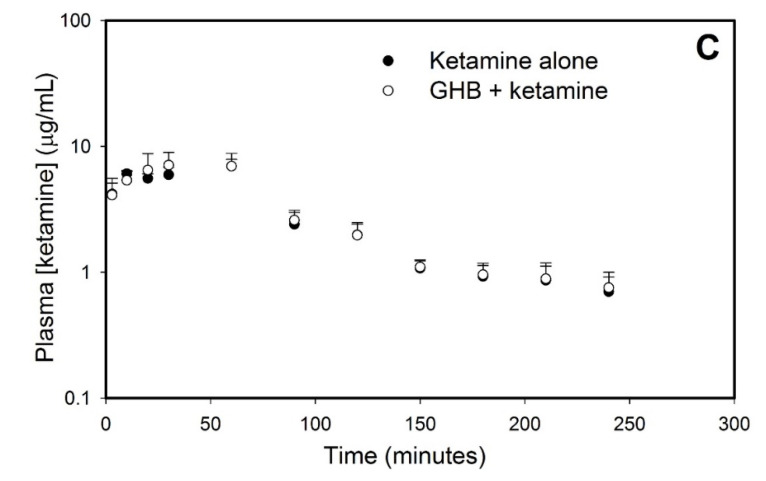
Plasma concentration-time profiles of GHB (600 mg/kg i.v.) and ketamine in the presence (*n* = 6) and absence (*n* = 5) of ketamine. (**A**) Plasma GHB concentrations, (**B**) Renal Clearance (CL_R_), Metabolic Clearance (CL_M_), and Total Clearance (CL_T_) of GHB in the presence and absence of ketamine, (**C**) Plasma ketamine concentrations in the presence and absence of GHB (*n* = 4 for both groups). Ketamine was administered 5 min before GHB administration as 6 mg/kg i.v. bolus followed by 1 mg/kg/min i.v. infusion for 60 min. Data are presented as mean ± S.D. * *p* < 0.001 compared to GHB alone using Student’s *t*-test.

Ketamine administration with GHB resulted in significantly higher GHB plasma concentrations when compared to GHB administration alone, as shown in Figure 2A. The total and metabolic clearance of GHB was found to be significantly decreased in the presence of ketamine with renal clearance remaining unchanged, as depicted in Figure 2B. However, there was no significant difference in the steady-state concentration of ketamine in the treatment group of animals receiving GHB and ketamine, when compared to the group of animals administered ketamine alone (Figure 2C).

In addition to the increase in GHB plasma exposure observed in the presence of ketamine, ketamine 0.287 mg/kg/min co-administration also resulted in a significant increase in GHB brain concentrations at steady state and this resulted in a significant increase in the GHB brain/plasma ratio when compared to GHB alone, as shown in Table 1. There was no significant effect of a low dose of ketamine (0.1 mg/kg/min) on GHB brain concentrations when compared to GHB alone.

#### 3.1.2. Effect of Ketamine on GHB Toxicodynamics

**Figure 3:**
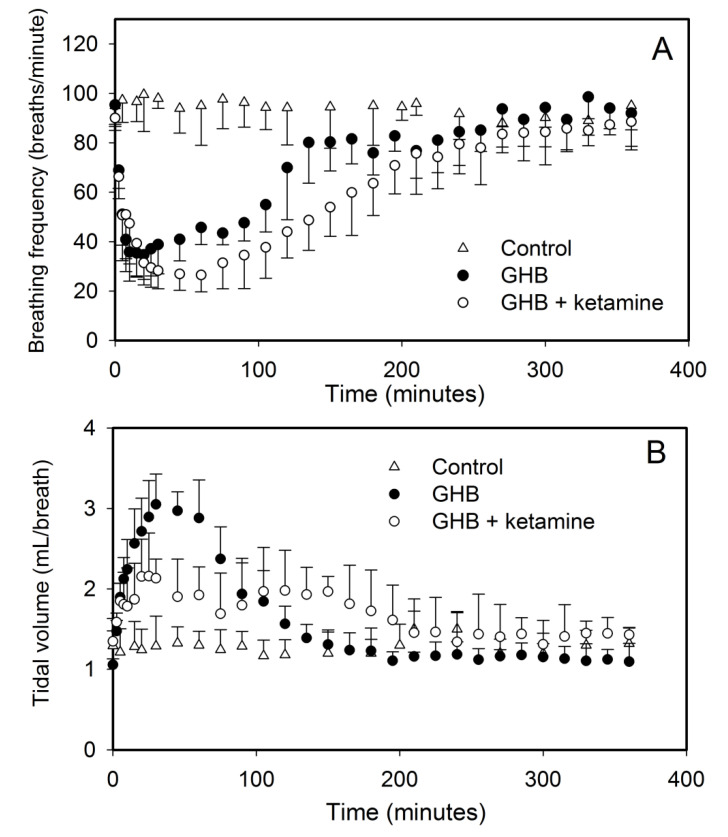

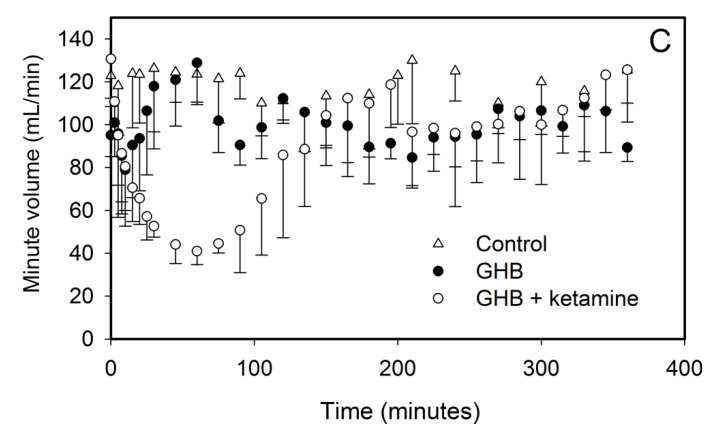
Effect of ketamine co-administration on GHB-induced respiratory depression. GHB 600 mg/kg i.v. was administered alone (*n* = 5) or with ketamine (6 mg/kg i.v. bolus + 1 mg/kg/min i.v. infusion for 60 min) (*n* = 6). Data presented as mean ± SD. Ketamine was administered 5 min before GHB administration. (**A**) Breathing frequency, (**B**) Tidal volume, (**C**) Minute volume (breathing frequency X tidal volume). *n* = 4 for control group.

**Figure 4:**
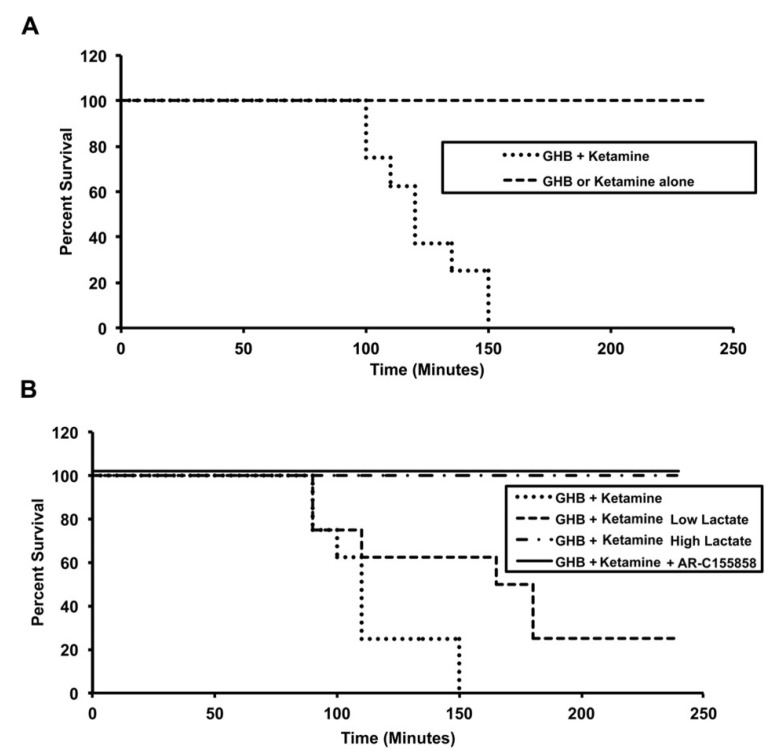
Effect of ketamine (**A**) and MCT inhibition (**B**) on fatality after administration of GHB. GHB was administered as 400 mg/kg i.v. bolus followed by 208 mg/kg/h i.v. infusion with or without ketamine (6 mg/kg i.v. bolus followed by 1 mg/kg/h i.v. infusion). L-Lactate (66 mg/kg i.v. bolus, followed by an infusion of 302.5 mg/kg/h (low dose) or 605 mg/kg/h (high dose) and AR-C155858 were administered 5 min after GHB-ketamine. *n* = 8 in each treatment group.

The effect of ketamine on GHB toxicodynamics was evaluated using the end points of sedation, respiratory depression, and fatality. The effect of ketamine on GHB-induced respiratory depression is displayed in Figure 3. GHB alone at 600 mg/kg produces a decrease in breathing frequency with a compensatory increase in tidal volume with minute volume (breathing frequency x tidal volume) remaining unchanged. Ketamine administration alone did not result in any significant respiratory depression and the results were highly variable due to increased mobility in these animals (data not shown). Ketamine co-administration with GHB resulted in a significant decline in breathing frequency as seen with an increase in AUEC and Emax for breathing frequency when compared to GHB alone (Table 2). Ketamine also prevented the compensatory increase in tidal volume as seen with a significant decrease in AUEC and E_max_ for tidal volume when compared to GHB alone (Figure 3B). This resulted in a significant decline in minute volume in the GHB-ketamine group when compared to GHB alone (Figure 3C). Administration of GHB (400 mg/kg bolus followed by a 208 mg/kg/h infusion) with ketamine (6 mg/kg bolus followed by a 1 mg/kg/min infusion) resulted in increased rates of fatality when compared with GHB alone, as shown in Figure 4. No death was observed in animals treated with either GHB or ketamine alone.

**Figure 5:**
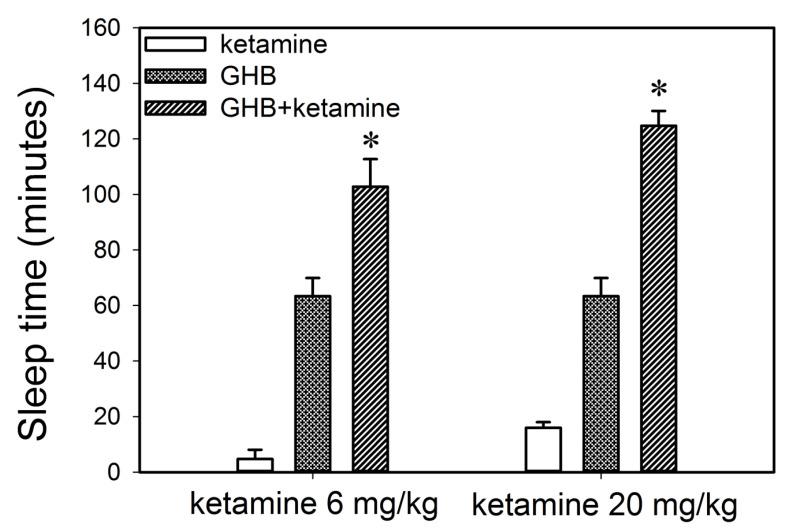
Effect of ketamine co-administration on the sedative effect of GHB. GHB (400 mg/kg i.v.) was administered with (*n* = 5) or without ketamine (6 or 20 mg/kg i.v.) (*n* = 4 for both groups). *n* = 3 for ketamine alone. Sleep time was measured as the difference between the loss and return of righting reflex. One-way analysis of variance followed by Tukey’s post-hoc test was used to determine statistically significant differences in sleep time between different treatment groups. Data presented as mean ± SD, *n =* 3–6. * *p* < 0.05 significantly different from GHB or ketamine alone.

Co-administration of ketamine with GHB also resulted in a significant increase in sleep time as displayed in Figure 5 when compared to the group treated with either GHB or ketamine alone. The increase in sleep time was observed at both the ketamine doses (6 or 20 mg/kg) used in this study. Previous results in our laboratory have demonstrated that there is a direct relationship between plasma GHB concentration and offset of sedative/hypnotic effect. Therefore, to further investigate the relationship between GHB concentration and its sedative/hypnotic effect in the presence of ketamine, GHB concentrations were assessed in plasma and brain (whole brain) at RRR (offset of effect) in animals treated with GHB in combination with ketamine (6 or 20 mg/kg). At both these ketamine doses, GHB plasma and brain concentrations at RRR were not significantly different when compared to GHB alone demonstrating that the concentration-effect relationship of GHB with its sedative effects is maintained in the presence of ketamine (Table 3). However, GHB brain/plasma ratio at RRR was significantly higher following the administration of ketamine, when compared to GHB alone, consistent with our results examining GHB partitioning at steady state, as described above.

**Figure 6:**
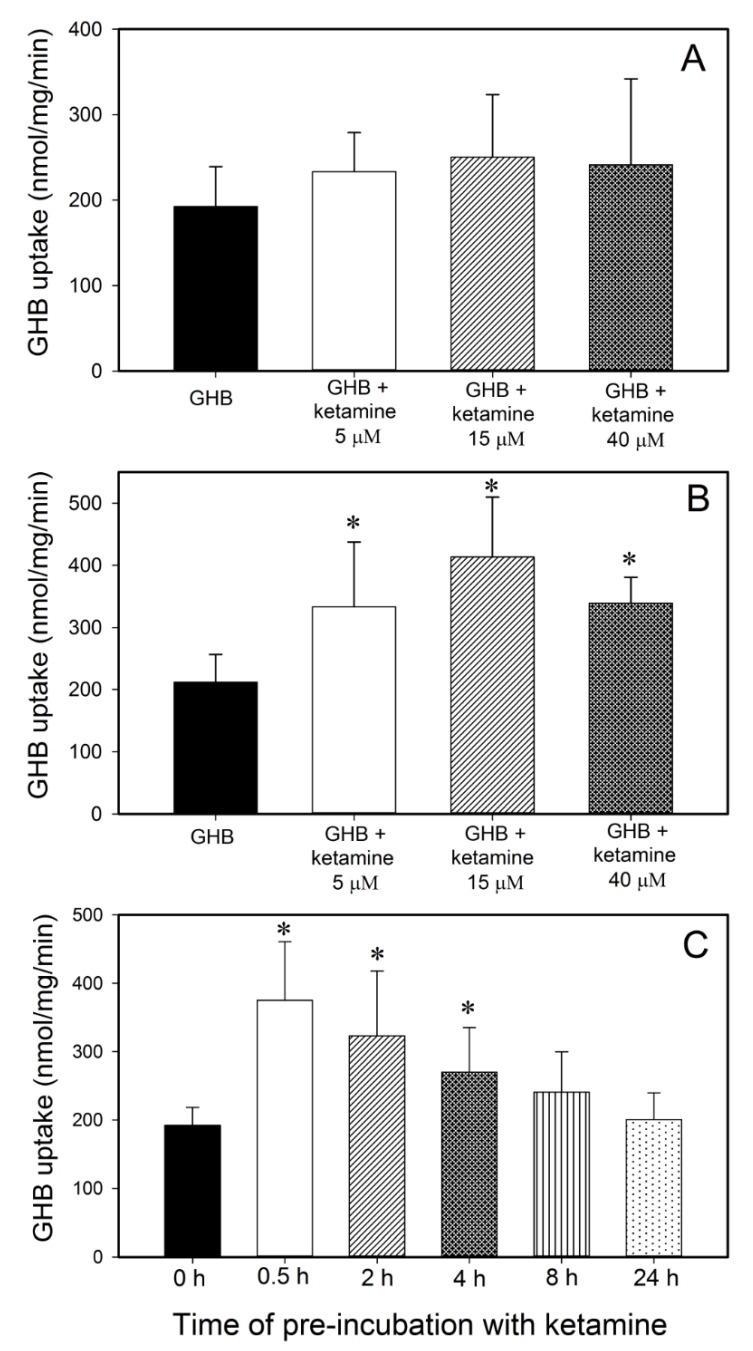
Effects of ketamine (5, 15, and 40 µM) on the uptake of GHB (10 mM) in RBE4 cells. (**A**) No-pre-incubation with ketamine, (**B**) 2 h pre-incubation with ketamine at 37 °C, (**C**) Pre-incubation with 40 µM ketamine for 0.5, 2, 4, 8, and 24 h at 37 °C. Data presented as mean ± SD of three sets of studies conducted in triplicate. One-way analysis of variance followed by Tukey’s post-hoc test was used to determine statistically significant differences in sleep time between different treatment groups. * *p* < 0.05 significantly from GHB alone.

Effect of ketamine on GHB uptake in vitro. Since we observed an increase in GHB brain/plasma ratio when animals were administered GHB in combination with ketamine, we examined the effects of ketamine on the uptake of 10 mM GHB in RBE4 cells, an in vitro model of rat blood-brain barrier (BBB). This concentration of GHB was used since it is similar to the GHB plasma concentrations at steady state seen after the intravenous administration of GHB (400 mg/kg bolus followed by 208 mg/kg/h infusion). The effects of ketamine were studied either after a 2 h ketamine pre-incubation or without ketamine pre-incubation when ketamine was only present in the uptake buffer with [^3^H]GHB. As seen in Figure 6A, there was no significant change in GHB uptake in the presence of ketamine without pre-incubation with ketamine. However, following a 2 h pre-incubation with ketamine (5, 15 or 40 µM), GHB uptake was significantly increased when compared with GHB alone (Figure 6B). These results further support our in vivo findings. In addition, we also examined the effects of various pre-incubation times (0.5, 2, 4, 8 and 24 h pre-incubation with 40 µM ketamine) on GHB uptake and found significant increases in GHB uptake following 0.5, 2, and 4 h pre-incubation with ketamine, with no significant changes observed at longer pre-incubation times (Figure 6C).

**Figure 7:**
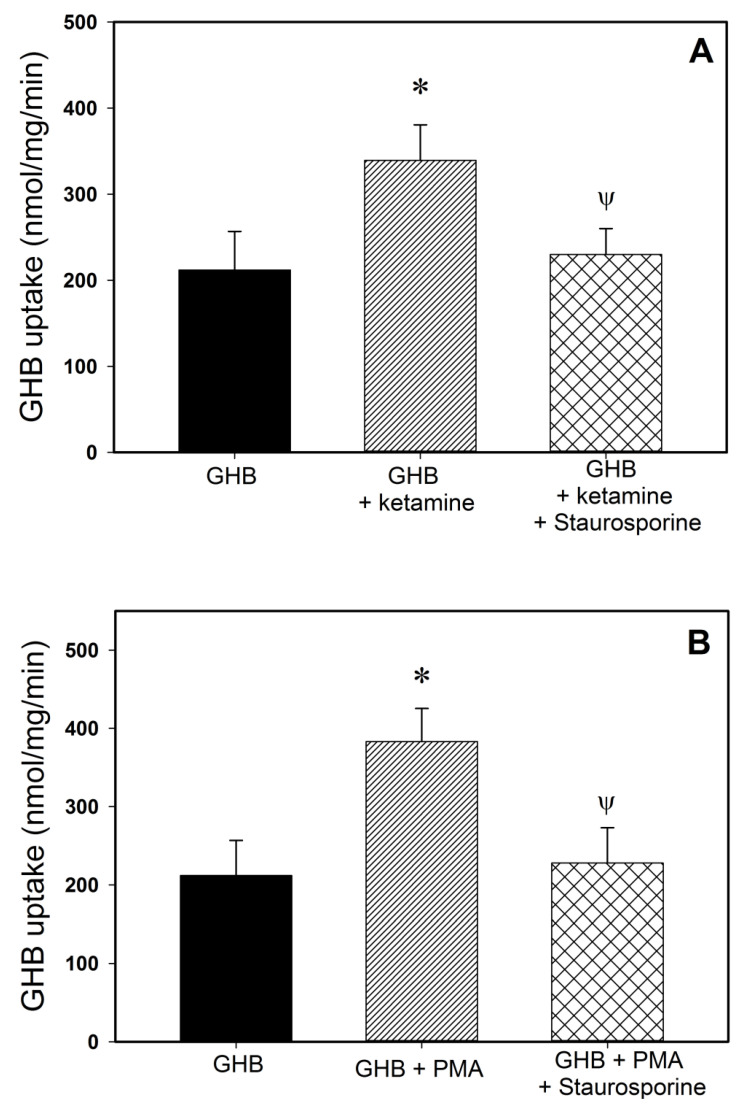
Effect of ketamine and PMA on the uptake of GHB in RBE4 cells in the presence and absence of the PKC inhibitor, staurosporine. (**A**) ketamine, (**B**) PMA. Cells were pre-incubated with ketamine (40 µM) or PMA (100 nM) in the presence and absence of staurosporine at 37 °C. Data presented as mean ± SD of three sets of studies conducted in triplicate. One-way analysis of variance followed by Tukey’s post-hoc test was used to determine statistically significant differences in sleep time between different treatment groups. * *p* < 0.05 significantly different from GHB alone; ^Ψ^ *p* < 0.05 significantly different from GHB + ketamine or GHB + PMA.

Pre-treatment of the cells with a PKC activator, PMA resulted in a significant increase in GHB uptake, similar to that observed with ketamine as shown in Figure 7. To further investigate the involvement of PKC in the increase in GHB uptake observed in the presence of ketamine, we further studied the effect of PMA and ketamine on GHB uptake in the presence and absence of a nonspecific PKC inhibitor, staurosporine (Figure 7). Pre-incubation of the cells with ketamine in the presence of staurosporine resulted in no increase in GHB uptake and uptake was similar to the GHB alone control. A significant increase in GHB uptake was observed in cells pre-treated with PMA; however, with co-incubation with staurosporine, no increase in GHB uptake was observed, similar to the effects observed with ketamine and staurosporine.

### 3.2. Potential Treatment Strategies for Overdose

#### 3.2.1. Monocarboxylate Transporter Inhibition

**Figure 8:**
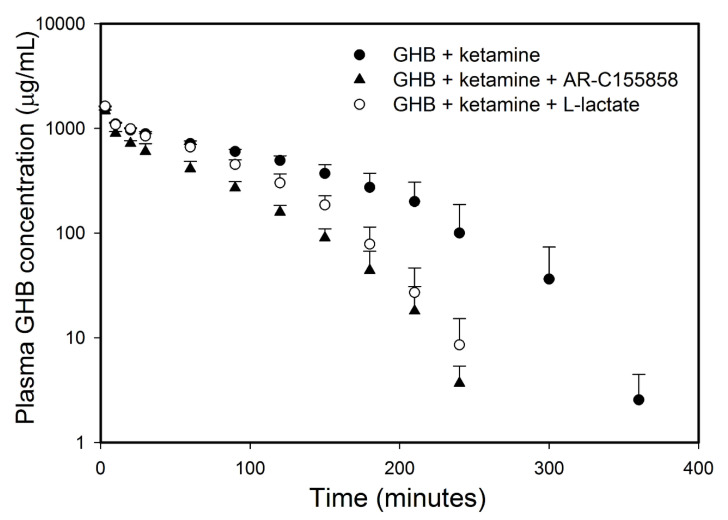
Effect of MCT inhibition on GHB plasma concentration-time profile in the presence of ketamine. GHB (600 mg/kg i.v. bolus) and ketamine (6 mg/kg i.v. bolus followed by 1 mg/kg/min i.v. infusion for 60 min) were administered without (*n* = 6) or with L-lactate (*n* = 4) or AR-C155858 (*n* = 4). L-lactate was administered as 66 mg/kg i.v. bolus plus 302.5 mg/kg/h i.v. infusion 5 min after GHB-ketamine administration and continued until 6 h. AR-C155858 was administered as 1 mg/kg i.v. bolus 5 min after GHB-ketamine administration. Data presented as mean ± SD.

Two MCT inhibitors, L-lactate and ARC-155858 were used to study the effects of MCT inhibition on GHB toxicokinetics and toxicodynamics (sedation, respiratory depression, and fatality) in the presence of ketamine. L-lactate (66 mg/kg i.v. bolus followed by 302.5 mg/kg/h i.v. infusion) and AR-C155858 (1 mg/kg i.v. bolus) resulted in a significant decrease in GHB plasma concentrations when compared to animals treated with GHB-ketamine (Figure 8). There was also a significant increase in GHB renal and total clearance when compared to the GHB-ketamine group as displayed in Table 4. In the AR-C155858 treated group, there was also a significant increase in GHB metabolic clearance when compared to GHB-ketamine. Both L-lactate and AR-C155858, administered 5 min after GHB-ketamine administration, significantly reduced GHB brain concentrations and GHB brain/plasma ratio at steady state when compared to GHB-ketamine (Table 1). GHB plasma steady-state concentrations were, however, only decreased by AR-C155858 treatment and no changes were observed with L-lactate.

**Figure 9:**
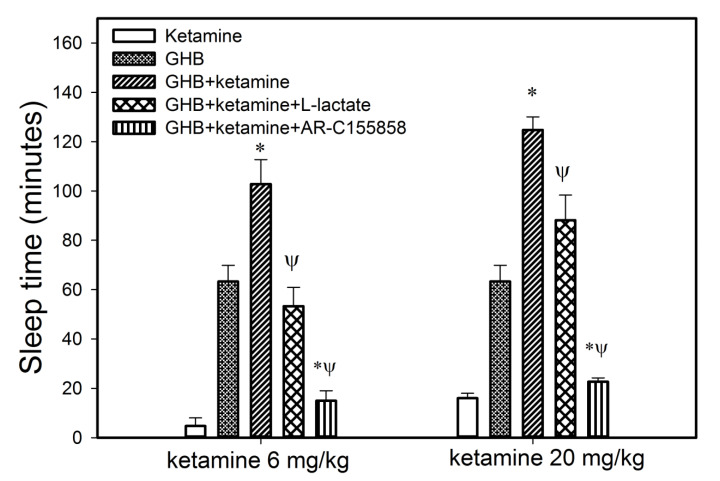
Effect of MCT inhibition on the sedative effect of GHB in the presence of ketamine. GHB (400 mg/kg i.v.) and ketamine (6 or 20 mg/kg i.v.) were administered with or without L-lactate or AR-C155858. L-lactate was administered as 66 mg/kg i.v. bolus plus 302.5 mg/kg/h i.v. infusion 5 min after GHB-ketamine administration and continued until animals were euthanized at RRR. AR-C155858 was administered as 1 mg/kg i.v. bolus 5 min after GHB-ketamine administration. Sleep time was measured as the difference between the loss and return of righting reflex. One-way analysis of variance followed by Tukey’s post-hoc test was used to determine statistically significant differences in sleep time between different treatment groups. Data presented as mean ± SD, *n* = 5 for GHB alone, *n* = 3 for ketamine alone, *n* = 4 for GHB + Ketamine 6 mg/kg, *n* = 4 for GHB + Ketamine 20 mg/kg, *n* = 4 for GHB + Ketamine 6 mg/kg + L-lactate, *n* = 4 for GHB + Ketamine 20 mg/kg + L-lactate, *n* = 3 for GHB + Ketamine 6 mg/kg + AR-C155858, *n* = 3 for GHB + Ketamine 20 mg/kg + AR-C155858. * *p* < 0.05 significantly different from GHB alone. ^Ψ^ significantly different from GHB + ketamine.

**Figure 10:**
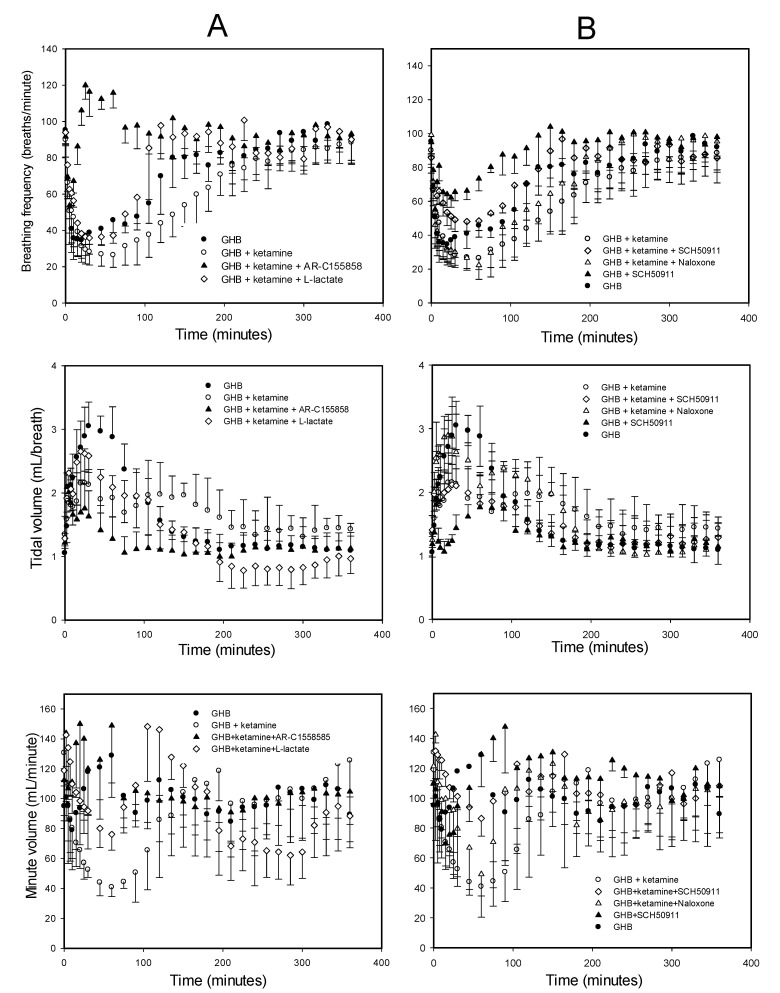
Effect of potential treatment strategies (**A**) MCT inhibition, and (**B**) Specific receptor antagonism on GHB-induced respiratory depression in the presence of ketamine. GHB (600 mg/kg i.v. bolus) and ketamine (6 mg/kg i.v. bolus followed by 1 mg/kg/min i.v. infusion) with or without MCT inhibitors, L-lactate (66 mg/kg i.v. bolus plus 302.5 mg/kg/h i.v. infusion), or AR-C155858 (1 mg/kg i.v. bolus), GABAB receptor antagonist, SCH50911 (10 mg/kg i.v. bolus) or µ-opioid receptor antagonist, naloxone (2 mg/kg i.v. bolus). The treatment strategies were administered 5 min after GHB-ketamine administration. Data presented as mean ± S.D. *n* = 5 for GHB alone, *n* = 6 for GHB + Ketamine, *n* = 4 for GHB + Ketamine + L-lactate, *n* = 3 for GHB + Ketamine + AR-C155858, *n* = 3 for GHB + Ketamine + SCH50911, *n* = 3 for GHB + Ketamine + naloxone.

Treatment with both L-lactate and AR-C155858 resulted in a significant decrease in sleep time when compared to the GHB-ketamine group at both ketamine 6 and 20 mg/kg doses (Figure 9). There was a 3-fold greater reduction in sleep time with AR-C155858 when compared to L-lactate. The respiratory depression studies showed that treatment with L-lactate resulted in a significant improvement in breathing frequency and minute volume (as seen with a decrease in AUEC for breathing frequency and minute volume) when compared to GHB-ketamine (Figure 10A). However, there was no change in tidal volume with L-lactate treatment compared to GHB-ketamine. AR-C155858 treatment resulted in a significant and rapid improvement in breathing frequency when compared to GHB-ketamine, as seen with a significant decrease in AUEC and reversal of the breathing frequency to baseline values within 20 min after GHB-ketamine administration. In addition, AR-C155858 completely reversed the decline in minute volume which was seen with the combination of GHB and ketamine. Administration of L-lactate (66 mg/kg i.v. bolus followed by 302.5 mg/kg/h i.v. infusion) decreased the rate of fatality observed with GHB-ketamine co-administration whereas a higher dose of L-lactate (66 mg/kg i.v. bolus followed by 605 mg/kg/h i.v. infusion) was needed to completely prevent fatality in these animals as shown in Figure 4B. Administration of AR-C155858 (1 mg/kg i.v. bolus) completely prevented fatality in the GHB-ketamine treated animals.

#### 3.2.2. Specific Receptor Antagonism

We also evaluated the effects of GABA_B_ receptor antagonist (SCH50911) and µ-opioid receptor antagonist (naloxone) on GHB-induced respiratory depression in the presence of ketamine. Treatment with SCH50911 (10 mg/kg) resulted in a significant improvement in breathing frequency and minute volume when compared to the GHB-ketamine group (Figure 10B). The compensatory increase in tidal volume was also absent in the SCH50911-treated group. However, SCH50911 had greater effects in the animals treated with GHB alone when compared to those treated with the combination of GHB and ketamine. Naloxone (2 mg/kg), on the other hand, had no effects on GHB-induced respiratory depression in the presence of ketamine. The combination of naloxone and SCH50911 provided no further improvement in respiratory depression when compared to the animals treated with SCH50911 alone.

## 4. Discussion

GHB is widely abused as a recreational drug alone as well as co-ingested with other commonly used club drugs, including ketamine. Considering the high frequency of co-ingestion of GHB with ketamine, the toxicokinetic/toxicodynamic interactions need to be characterized. Furthermore, potential treatment strategies also need to be studied under the most prevalent conditions of GHB abuse, i.e., in the presence of other club drugs. In the current study, we attempted to study the effects of ketamine on the TK/TD of GHB by using clinically relevant end points such as sedation, respiratory depression, and fatality. We also studied the effect of MCT inhibition and specific receptor antagonism on TK/TD of GHB when administered in the presence of ketamine to determine their usefulness as potential treatment strategies for overdose of this club drug combination.

Our toxicokinetic studies indicate that plasma exposure of GHB is significantly increased in the presence of ketamine when compared to GHB alone and metabolic and total clearance is significantly decreased while renal clearance remains unchanged. Ketamine concentrations were, however, not affected by GHB and they remained similar in the animals treated with ketamine alone compared to GHB-ketamine. Ketamine plasma concentrations associated with fatalities have been reported in the range of 1.8–27.4 µg/mL [30,31,32]. Therefore, the ketamine concentrations used in study were ~7 µg/mL to mimic ketamine clinical overdose concentrations. GHB exhibits capacity-limited metabolism and is characterized by Michaelis Menten kinetics [10]. The metabolic pathways involved in the metabolism of GHB are complex with involvement of both mitochondrial and cytosolic enzymes. The rate-limiting step in the metabolism of GHB is the formation of succinic semialdehyde (SSA), through a process which involves GHB transhydrogenase in the mitochondria and the GHB dehydrogenase in cytosol [33]. Ketamine, via a reduction in mitochondrial membrane potential as well as reduction in NADH dehydrogenase activity, can produce mitochondrial dysfunction, which then consequently affects ATP synthesis [34]. Ketamine has also been shown to potentiate the hepatotoxicity of cocaine as demonstrated by an increase in serum alanine aminotransferase and aspartate aminotransferase levels [35]. These mechanisms could be involved in the decrease in GHB metabolic clearance in the presence of ketamine observed in our study. Interestingly, ketamine has been reported to noncompetitively inhibit the glucuronidation of morphine and codeine [36,37] exhibiting inhibitory effects on UGT2B4, 2B7, and 2B15. The effects of ketamine on individual enzymes in the GHB metabolic pathway have not been studied and needs further evaluation based on our in vivo findings.

Additionally, we have demonstrated that brain concentrations of GHB at steady state are significantly increased in the presence of ketamine (0.287 mg/kg/min), due to increased blood-brain partitioning of GHB (Table 1), as assessed by the brain/plasma ratio of GHB in rats treated with GHB-ketamine. No changes were observed with the lower dose of ketamine used in this study. Our in vitro studies in RBE4 cells also show an increase in GHB uptake after pre-incubation with ketamine for up to 4 h, further supporting this in vivo finding. MCT1 is the only isoform present at the BBB [38] and is therefore responsible for the transport of GHB into the brain [14,15]. Our data suggest that ketamine may affect the short-term regulation of MCT1 which may involve its trafficking into the plasma membrane. The short-term regulation of MCTs and its underlying molecular mechanisms are poorly understood. CD147 (basigin) which is an ancillary protein, has been suggested in the literature as being necessary for trafficking, localization, as well as functional expression of MCT1 at the plasma membrane, where the two proteins are known to be tightly bound together [39]. The mechanisms responsible for the increased brain uptake may relate to either post-transcriptional regulation of MCT1, or increased trafficking of MCT1 protein to the plasma membrane, or changes in MCT1-basigin complex’s expression or stability. Narumi et al. reported that uptake of L-lactate (an MCT substrate) is stimulated by a 24 h pre-incubation with phorbol 12-myristate (PMA), a protein kinase C (PKC) activator, via an increase of the transport Vmax, and this is associated with an increased MCT1 protein expression in skeletal muscle cells [40]. These authors did not observe any effect following a 1-h exposure to PMA on the uptake of L-lactate and concluded that PMA has no effect on short-term regulation of MCT1 such as phosphorylation or recruitment to the plasma membrane in skeletal muscle cells. However, our results have shown that a 2 h pre-incubation with PMA significantly increases GHB uptake in RBE4 cells, similar to that observed with ketamine (Figure 10B). In addition, the increase in GHB uptake by PMA and ketamine are significantly inhibited by a PKC inhibitor, staurosporine (Figure 10). PKC is found in almost all cell types. Kudoh et al. reported a stimulatory effect of ketamine on PKC [41]. Therefore, the results of this study suggest that ketamine increases the uptake of GHB in RBE4 cells through short-term regulation mechanisms such as effects on MCT1 trafficking to the membrane. The discrepancy between our results and that of Narumi et al., where effects were observed only over longer pre-incubation times, could be attributed to different isozymes of MCT with distinct biochemical properties and cell-specific expression contributing to cell- and organ-specific effects (skeletal muscle vs. brain cells). Our results are also consistent with previous reports of direct modulation of MCT1 kinetic function by a cAMP-mediated protein kinase A signaling pathway in RBE4 cells [42]. This would be consistent with the findings for the organic anion transporter 3 (OAT3) where PKC has both short-term and long-term effects on the trafficking and stability of OAT3 [43]. Therefore, our results suggest that the effects of ketamine on GHB uptake may involve short-term regulation of MCT1 through activation of PKC; this potential regulatory mechanism requires further confirmation.

Previous studies in rodents have demonstrated that GHB produces dose-dependent sedative/hypnotic effects which are mediated by GABA_B_ receptors [21,29]. It has also been shown that GHB concentrations in the plasma and brain correlate with its sedative effect [29]. Ketamine also produces a dose-dependent sedation in rodents which is mediated by the HCN1 pacemaker channels that underlie a neuronal hyperpolarization-activated cationic current [24]. Therefore, we studied the effect of ketamine on the sedative effects of GHB using RRR as an end point. Our sedation studies demonstrate that ketamine significantly increases the sedative effects of GHB, similar to reports with GHB and ethanol [6,7], and suggest a synergistic interaction between GHB and ketamine. There was no significant difference in plasma and brain GHB concentrations at RRR when compared to GHB alone further suggesting that the concentration-sedative effect relationship of GHB (as seen with GHB alone) is maintained in the presence of ketamine. However, the brain/plasma ratio of GHB at RRR was significantly increased in the presence of ketamine at both doses (6 or 20 mg/kg) when compared to GHB alone, indicating increased GHB brain partitioning following ketamine administration. This was further confirmed by the significant increase in GHB steady-state–state brain/plasma ratio in the presence of ketamine as discussed above. These data thus suggest that the increase in GHB-induced sleep time observed in the presence of ketamine may be partly mediated by the increase in GHB partitioning into its effect site in the brain and may involve effects of ketamine on MCT1 regulation.

In a recent report in 226 cases of GHB-associated fatalities, the most common cause of death was cardio-respiratory arrest [3]. Respiratory depression has also been reported with nonfatal cases of GHB intoxication [5]. Recent studies in our laboratory have shown that GHB can also cause dose-dependent respiratory depression in rats [19]. GHB is known to bind to both GHB and GABA_B_ receptors, with its pharmacological effects of sedation, hypothermia and respiratory depression mediated by binding to GABA_B_ receptors in the brain [19,21,22]. Ketamine is a non-competitive *N*-methyl-d-aspartate receptor (NMDA) receptor antagonist which accounts for most of its anesthetic effects. Intraperitoneal administration of ketamine has been shown to cause significant respiratory depression in mice which was completely abolished in μ-opioid receptor knockout mice [25]. Measurement of respiration in human volunteers after intravenous ketamine administration also showed a log-linear dose related depression [26]. This suggests that ketamine produces respiratory depression through mechanisms different from that of GHB and its respiratory effects are mediated by binding to µ-opioid receptors. Ketamine has also shown to potentiate the respiratory depression induced by opioids when administered at subanesthetic doses in rats [28]. Koek et al. have shown that NMDA antagonists such as ketamine and phencyclidine can enhance the cataleptic effects of GHB, but not of baclofen (a GABA_B_ receptor agonist), and they do so in the order of their relative potencies as NMDA receptor antagonists [27]. However, NMDA receptor binding has not been associated with respiratory depression for ketamine. Therefore, in the present study, we assessed the effects of ketamine on GHB-induced respiratory depression, and the role of GABA_B_ and µ-opioid receptors in this toxic end point.

The results of the present study demonstrate that ketamine significantly lowers the breathing frequency when compared to GHB alone. In addition, ketamine prevented the compensatory increase in tidal volume, normally observed with GHB alone, which resulted in a significant decline in minute volume in the animals treated with GHB-ketamine. It is interesting to note that GHB alone does not result in any reduction in minute volume at the dose used in this study due to the compensatory increase in tidal volume produced with the administration of GHB [19]. Ketamine concentrations were maintained at 7 µg/mL up to 1 h in this study. However, when high GHB concentrations were maintained with similar steady-state concentrations of ketamine for a longer time, we observed fatality in all the animals in this treatment group, most likely due to enhancement in the respiratory depressant effects of GHB in the presence of ketamine. To our knowledge, this is the first report demonstrating that ketamine at high concentrations can result in an increased risk of respiratory depression and fatality when combined with GHB.

One of the proposed treatment strategies for GHB overdose is GABA_B_ receptor antagonism. We have previously shown in our laboratory that GABA_B_ receptor antagonism can also serve as a potential treatment strategy for GHB overdose by blocking respiratory depression. However, the effectiveness of GABA_B_ receptor antagonism in treating GHB overdose when it is co-ingested with ketamine currently remains unknown. Therefore, we tested the effect of SCH50911 (a potent GABA_B_ receptor antagonist) on GHB-induced respiratory depression in the presence of ketamine. Our results demonstrate that SCH50911 can improve GHB-induced respiratory depression when it is co-administered with ketamine. Interestingly, we observed a greater effect of SCH50911 in the animals treated with GHB alone (data not shown) when compared to the animals treated with GHB-ketamine, suggesting the involvement of receptors in addition to GABA_B_. However, the µ-opioid receptor antagonist, naloxone (an approved antidote for opioid overdose), alone or in combination with GABA_B_ receptor antagonism, had no effect on GHB/ketamine-induced respiratory depression. This data suggest that the potentiating effects of ketamine are not mediated by µ-opioid receptors. Naloxone has been reported to shown minimal effects on GHB-induced coma in overdose in humans [44], consistent with our findings. There is also a possibility of the involvement of other receptors including NMDA receptors in the observed toxicodynamic GHB-ketamine interaction. However, this was not evaluated in our studies as ketamine-induced respiratory depression was found to be completely abolished in µ-opioid receptor knockout mice [25].

Previous results in our laboratory have demonstrated the use of MCT inhibition as a potential treatment strategy for GHB overdose. L-lactate results in an increase in GHB renal and total clearance by inhibiting its MCT-mediated renal reabsorption [11,18]. Higher doses of L-lactate (resulting in concentrations above 5 mM) have also shown to decrease GHB brain extracellular concentrations in rats with no effects with lower L-lactate doses [20]. This study extends the use of MCT inhibition as treatment strategy for GHB overdose when it is co-administered with ketamine, representing a more clinically relevant scenario. We also studied the effects of a more potent MCT inhibitor, AR-C155858 (Ki ~2.3 nM for MCT1) on the TK/TD of this combination [45]. Both L-lactate and AR-C155858 treatments resulted in an increase in the renal as well as total clearance of GHB, when compared to the GHB-ketamine group. Interestingly, the brain/plasma ratio of GHB at steady state was significantly reduced in the presence of the MCT inhibitors when compared to GHB-ketamine. However, AR-C155858, but not L-lactate reduced the GHB brain/plasma ratio in comparison to GHB alone. This finding demonstrates that more potent inhibitors of MCT can result in both inhibition of GHB renal reabsorption and brain uptake, serving as potential candidates for overdose treatment strategies. Both L-lactate and AR-C155858 improved GHB-induced respiratory depression and sleep time in the presence of ketamine with AR-C155858 with 3-fold higher effects owing to its higher potency. In addition, a low dose of L-lactate provided partial improvement whereas a higher dose of L-lactate provided complete reversal of the fatality observed with GHB-ketamine. This is consistent with our previous studies with decreased brain/plasma ratio of GHB only at the higher doses of L-lactate [20].

## 5. Conclusions

We observed significant toxicokinetic interactions between the club drugs, GHB and ketamine. Ketamine co-administration with GHB resulted in decreased metabolic and total clearance of GHB with no effects on renal clearance. Ketamine also increased GHB brain/plasma ratio at steady state. Studies of GHB uptake in vitro were consistent with our in vivo findings suggesting that ketamine can alter the blood-brain barrier transport of GHB through a mechanism that may involve increased MCT1 expression, mediated by ketamine effects on PKC. GHB-induced respiratory depression, sedation, and lethality can be significantly increased with concurrent ketamine administration. Both MCT inhibition and GABA_B_ receptor antagonism can serve as potential treatment strategies for GHB overdose when it is co-ingested with ketamine; however, µ-opioid receptor antagonism using naloxone had no effects on the toxicity of GHB in the presence of ketamine.

## Figures and Tables

**Table 1:** Effect of ketamine co-administration on GHB blood-brain partitioning.

Treatment	C_plasma ketamine_ (µg/mL)	C_plasma GHB_ (mg/mL)	C_brain GHB_ (mg/g)	GHB Brain/Plasma Ratio
GHB alone	---	0.89 ± 0.05	0.21 ± 0.03	0.24 ± 0.02
GHB + ketamine 0.1 mg/kg/min	0.78 ± 0.05	0.92 ± 0.05	0.20 ± 0.03	0.21 ± 0.02
GHB + ketamine 0.287 mg/kg/min	2.26 ± 0.21	0.90 ± 0.07	0.33 ± 0.05 *	0.36 ± 0.05 *
GHB + ketamine 0.287 mg/kg/min + L-lactate	2.67 ± 0.47	0.84 ± 0.04	0.17 ± 0.02 ^Ψ^	0.20 ± 0.02 ^Ψ^
GHB + ketamine 0.287 mg/kg/min + AR-C155858	2.50 ± 0.30	0.37 ± 0.04 *^Ψ^	0.03 ± 0.004 *^Ψ^	0.08 ± 0.01 *^Ψ^

GHB 400 mg/kg i.v. bolus + 208 mg/kg/h i.v. infusion was administered alone (*n* = 7) or with ketamine (6 mg/kg i.v. bolus + 0.1 (*n* = 4) or 0.287 mg/kg/min i.v. infusion (*n* = 4)). L-lactate was administered as 66 mg/kg i.v. bolus plus 302.5 mg/kg/h i.v. infusion 5 min after GHB-ketamine administration and continued until animals were euthanized at steady state at 4 h (*n* = 4). AR-C155858 was administered as 1 mg/kg i.v. bolus 5 min after GHB-ketamine administration. Brain and plasma samples were obtained at 4 h (*n* = 3). One-way analysis of variance with Tukey’s post-hoc test was used to determine statistically significant differences. Data presented as mean ± SD. * Significantly different from GHB alone (*p* < 0.001); ^Ψ^ significantly different from GHB + ketamine (*p* < 0.001).

**Table 2:** Effect of ketamine and potential treatment strategies for the treatment of GHB-induced respiratory depression

Toxicodynamic Parameter	GHB (*n* = 5)	GHB + Ketamine (*n* = 6)	GHB + Ketamine L-lactate (*n* = 4)	GHB + Ketamine AR-C155858 (*n* = 4)	GHB + Ketamine SCH50911 (*n* = 3)	GHB + Ketamine Naloxone (*n* = 3)
Frequency AUEC (breaths)	5540 ± 1000	15,639 ± 1806 *	5933 ± 2300 ^Ψ^	320.3 ± 135 *^Ψ^	4534 ± 405 *^Ψ^	11,358 ± 3800
Frequency E_max_ (breaths/min)	31 ± 5	22.6 ± 4.5 *	34.5 ± 3.90 ^Ψ^	53.8 ± 7.31 *^Ψ^	47.9 ± 5.6 ^Ψ^	22.3 ± 8.32
Frequency T_d_ (min)	153 ± 12.5	326 ± 25.6 *	124 ± 18.9 ^Ψ^*	17.5 ± 2.90 *^Ψ^	140 ± 31.2 ^Ψ^	235 ± 45.8

GHB (600 mg/kg i.v. bolus) and ketamine (6 mg/kg i.v. bolus followed by 1 mg/kg/min i.v. infusion) with or without MCT inhibitors, L-lactate (66 mg/kg i.v. bolus plus 302.5 mg/kg/h i.v. infusion), or AR-C155858 (1 mg/kg i.v. bolus), GABAB receptor antagonist, SCH50911 (10 mg/kg i.v. bolus) or µ-opioid receptor antagonist, naloxone (2 mg/kg i.v. bolus). The treatment strategies were administered 5 min after GHB-ketamine administration. Data presented as mean ± S.D. One-way analysis of variance followed by Tukey’s post-hoc test was used to determine statistically significant differences in mean toxicodynamic parameters between groups. * *p* < 0.05 significantly different than GHB alone; ^Ψ^ *p* < 0.05 significantly different from GHB + ketamine.

**Table 3:** Effect of ketamine on GHB plasma and brain concentrations at RRR.

	C_plasma_ (µg/mL)	C_brain_ (µg/g)	GHB Brain/Plasma Ratio
GHB	379 ± 86.2	71.7 ± 9.89	0.19 ± 0.02
GHB + ketamine 6 mg/kg	277 ± 56.9	71.5 ± 8.47	0.26 ± 0.03 *
GHB + ketamine 20 mg/kg	293 ± 35.1	82.6 ± 6.25	0.28 ± 0.01 *

C_brain_, brain GHB concentration at RRR; C_plasma_, plasma GHB concentration at RRR. GHB (400 mg/kg i.v.) was administered with (*n* = 7) or without ketamine (6 or 20 mg/kg i.v.) (*n* = 4 for both groups). Animals were euthanized at RRR and brain and plasma collected. Data from 400 mg/kg alone GHB were used from a previous study [28]. One-way analysis of variance followed by Tukey’s post-hoc test was used to determine statistically significant differences between treatment groups. Data presented as mean ± SD, *n* = 3–6. * Significantly different from GHB alone (*p* < 0.05).

**Table 4:** Effect of MCT inhibition on toxicokinetics of GHB in the presence and absence of ketamine.

Parameter	GHB	GHB + Ketamine	GHB + Ketamine + AR-C155858	GHB + ketamine + L-Lactate
AUC (mg.min/mL)	101.9 ± 12.4	135.1 ± 14.4 *	66.8 ± 4.39 *	98.5 ± 5.73 ^Ψ^
CL_T_ (mL/min/kg)	6.00 ± 0.74	4.49 ± 0.55 *	9.01 ± 0.63 *	6.10 ± 0.34 ^Ψ^
CL_R_ (mL/min/kg)	1.68 ± 0.75	1.61 ± 0.29	4.10 ± 0.67 *	2.32 ± 0.38 ^Ψ^
CL_M_ (mL/min/kg)	4.31 ± 0.33	2.87 ± 0.28 *	5.04 ± 0.86 ^Ψ^	3.77 ± 0.45

GHB (600 mg/kg i.v. bolus) and ketamine (6 mg/kg i.v. bolus followed by 1 mg/kg/min i.v. infusion for 60 min) were administered without (*n* = 6) or with L-lactate (*n* = 4) or AR-C155858 (*n* = 4). L-lactate was administered as 66 mg/kg i.v. bolus plus 302.5 mg/kg/h i.v. infusion 5 min after GHB-ketamine administration and continued until 6 h. AR-C155858 was administered as 1 mg/kg i.v. bolus 5 min after GHB-ketamine administration. Data presented as mean ± SD. One-way analysis of variance followed by Tukey’s post-hoc test was used to determine statistically significant difference between treatment groups. * *p* < 0.001 significantly different than GHB alone; ^Ψ^ *p* < 0.001 significantly different than GHB + ketamine.

## Data Availability

Data is available from the corresponding author.
